# Fatigue strength of common tibial intramedullary nail distal locking screws

**DOI:** 10.1186/1749-799X-4-11

**Published:** 2009-04-16

**Authors:** Lanny V Griffin, Robert M Harris, Joseph J Zubak

**Affiliations:** 1 California Polytechnic State University, Biomedical and General Engineering, San Luis Obispo, CA, USA; 2 Director of Orthopaedic Trauma, Holston Valley Medical Center, Wellmont Health System, Kingsport, TN, USA; 3 Shannon Clinic Southwest, 4550 Sunset Dr. San Angelo, TX, USA

## Abstract

**Background:**

Premature failure of either the nail and/or locking screws with unstable fracture patterns may lead to angulation, shortening, malunion, and IM nail migration. Up to thirty percent of all unreamed nail locking screws can break after initial weight bearing is allowed at 8–10 weeks if union has not occurred. The primary problem this presents is hardware removal during revision surgery. The purposes of our study was to evaluate the relative fatigue resistance of distal locking screws and bolts from representative manufacturers of tibial IM nail systems, and develop a relative risk assessment of screws and materials used. Evaluations included quantitative and qualitative measures of the relative performance of these screws.

**Methods:**

Fatigue tests were conducted to simulate a comminuted fracture that was treated by IM nailing assuming that all load was carried by the screws. Each screw type was tested ten times in a single screw configuration. One screw type was tested an additional ten times in a two-screw parallel configuration. Fatigue tests were performed using a servohydraulic materials testing system and custom fixturing that simulated screws placed in the distal region of an appropriately sized tibial IM nail. Fatigue loads were estimated based on a seventy-five kilogram individual at full weight bearing. The test duration was one million cycles (roughly one year), or screw fracture, whichever occurred first. Failure analysis of a representative sample of titanium alloy and stainless steel screws included scanning electron microscopy (SEM) and quantitative metallography.

**Results:**

The average fatigue life of a single screw with a diameter of 4.0 mm was 1200 cycles, which would correspond roughly to half a day of full weight bearing. Single screws with a diameter of 4.5 mm or larger have approximately a 50 percent probability of withstanding a week of weight bearing, whereas a single 5.0 mm diameter screw has greater than 90 percent probability of withstanding more than a week of weight bearing. If two small diameter screws are used, our tests showed that the probability of withstanding a week of weight bearing increases from zero to about 20 percent, which is similar to having a single 4.5 mm diameter screw providing fixation.

**Conclusion:**

Our results show that selecting the system that uses the largest distal locking screws would offer the best fatigue resistance for an unstable fracture pattern subjected to full weight bearing. Furthermore, using multiple screws will substantially reduce the risk of premature hardware failure.

## Introduction

Tibial fractures are the most common long bone injury. Various methods of managing tibial fractures have been described in the literature over the years, ranging from plaster, functional bracing, compression plating external fixation and intramedullary (IM) nailing [1-8].

Kuntscher first described the technique of IM nailing femur fractures in the German [9] and later in the American literature [10]. Since its introduction, IM nailing has become a reliable treatment for a wide range of long bone fractures. Revisions to Kuntscher's original technique and nail design have been made by several authors to accommodate the shape of the tibial IM canal [11]. With the introduction of interlocking by Klemm and Schellman in 1972, the indications for IM nailing were expanded [12]. IM nailing has now has become the treatment of choice for managing tibial fractures [13-15].

While IM nailing is a significant advancement in fracture treatment, hardware failure is a complication of static IM nailing [16-18]. Premature failure of either the nail and/or locking screws with unstable fracture patterns may lead to angulation, shortening, malunion, and IM nail migration [16]. This can occur in cases of a non-compliant patient or an overly aggressive rehabilitation protocol. Thirty percent of all unreamed nail locking screws can break after initial weight bearing is allowed at 8–10 weeks if union has not occurred [16]. The primary problem this presents is hardware removal during revision surgery [16].

The purposes of our study was to evaluate the relative fatigue resistance of distal locking screws and bolts from representative manufacturers of tibial IM nail systems, and develop a relative risk assessment of screws and materials used. Evaluations included quantitative and qualitative measures of the relative performance of these screws.

## Methods

Tibial locking screws/bolts were obtained from the manufacturers listed in Table 1. Fatigue tests were conducted using a servohydraulic materials testing system (Instron, Model 8521s, Canton, MA, U.S.A.) equipped with a twenty-five (25) kN fatigue rated load cell. The tests were conducted in load control mode with a sinusoidal load profile with a peak compressive load of 2400 N and a minimum compressive load of 100 N, simulating peak loads of joint reaction forces of a normal gait cycle of a seventy-five kg individual with full weight bearing [19]. The fatigue test was conducted until complete fracture of the screw occurred or until one million cycles were reached.

**Table 1:** Locking screws that were evaluated for fatigue life

*Manufacturer*	*Diameter (mm)*	*Material*
Ace	4.5	Ti-6Al-4V

Biomet	4.0	Ti-6Al-4V
	5.0	

Howmedica-Alta	3.7	TMZ
	5.0	

Russell-Taylor	4.5	316 SS
	5.0	

Synthes	3.9	Ti-6Al-7Nb
	4.9	

S&N – Trigen	5.0	Ti-6Al-4V

**Figure 1:**
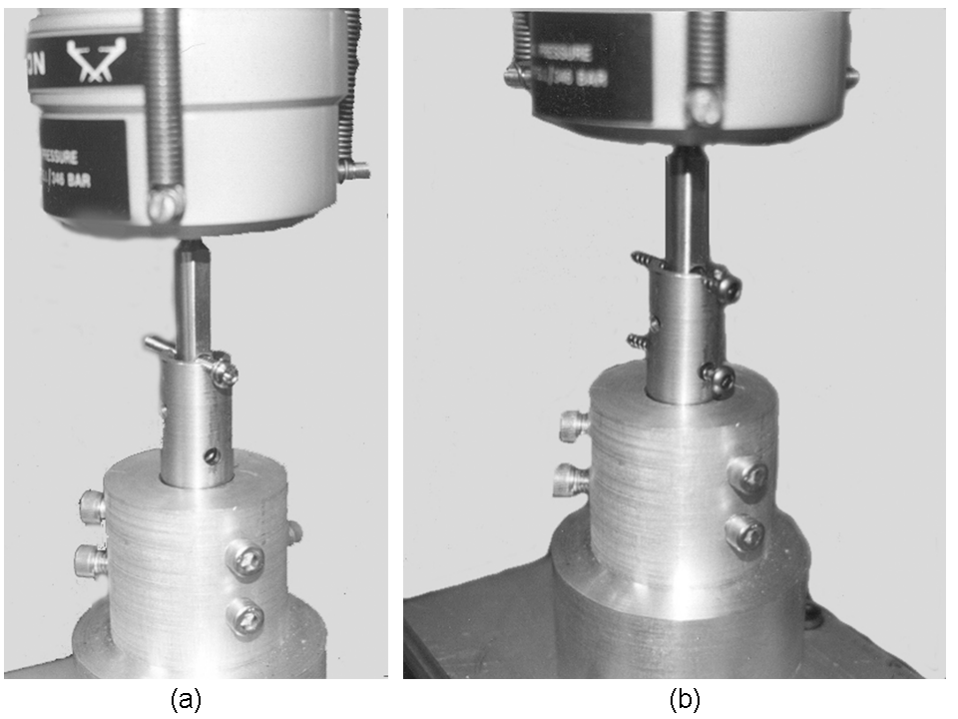
**The experimental configuration for fatigue tests that used (a) one screw, (b) two screws**.

Ten screws of each type listed in Table 1 were tested in single screw configuration. Tests were conducted at twenty Hz using a custom fixture that simulated the distal end of a 11.5 mm diameter intramedullary nail (Figure 1a). We used two fixtures, which produce a simply supported bending condition with a span of 15.5 mm. An additional twenty 3.9 mm diameter locking screws (Synthes) tested the effect of multiple screws on the fatigue life of the IM nail system. The screws were oriented in a parallel loading configuration as shown in Figure 1b. All other factors remained the same.

For the purposes of analysis, we classified the screws as large (4.9 mm or 5.0 mm), medium (4.5 mm) and small (4.0 mm or less). The two small screw configuration was included in the medium group. Regression with life data was performed using a Minitab Statistical Software package (Minitab 15, Minitab Inc, State College, PA). This regression was performed for various probability distributions commonly utilized in fatigue life analysis: smallest extreme value, Weibull, exponential, normal, lognormal, logistic, and loglogistic. Data of models that ran-out to one million cycles without failure were defined as right-censored and included in this analysis. One million cycles was assumed to be equivalent to one year of loading. The probability plot for standardized residuals in addition to an adjusted Anderson-Darling statistic was calculated for each distribution and the best-fitting distribution was selected. Finally, using best fitting distribution, a table of survival probabilities was generated for the various models tested at 2500 cycles (approximately one day), 20000 cycles (approximately one week) and 50000 cycles (2.5 weeks).

Since the distributions are not normal, we used the 50 percent probability of survival as being analogous to the average life and performed post-hoc Tukey's tests to determine statistically significant differences in fatigue life

## Results

**Figure 2:**
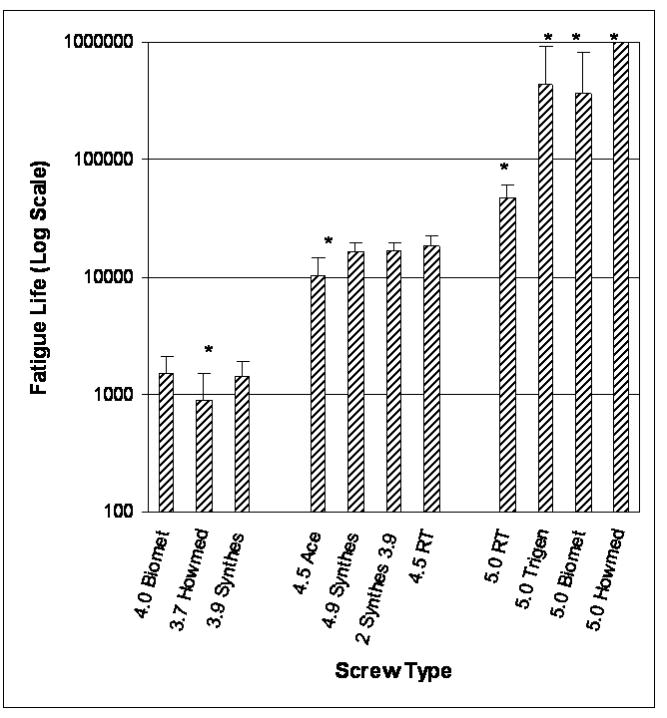
**Fatigue life results for the locking screws tested**. Asterisks (*) denote significant difference in mean life within the group (small, medium, or large diameter) at p < 0.05.

The results of the fatigue life studies are presented in Figure 2. Screw diameter was the main determinant of fatigue resistance. All screws smaller than 4.0 mm diameter perform similarly, failing around 1200 cycles or less; however, the Howmedica 3.7 mm diameter Alta™ screw failed below 1000 cycles and was statistically different from the other screws. For the two small diameter screws in parallel, the fatigue life was no different than using one larger diameter screw in the medium size grouping.

**Figure 3:**
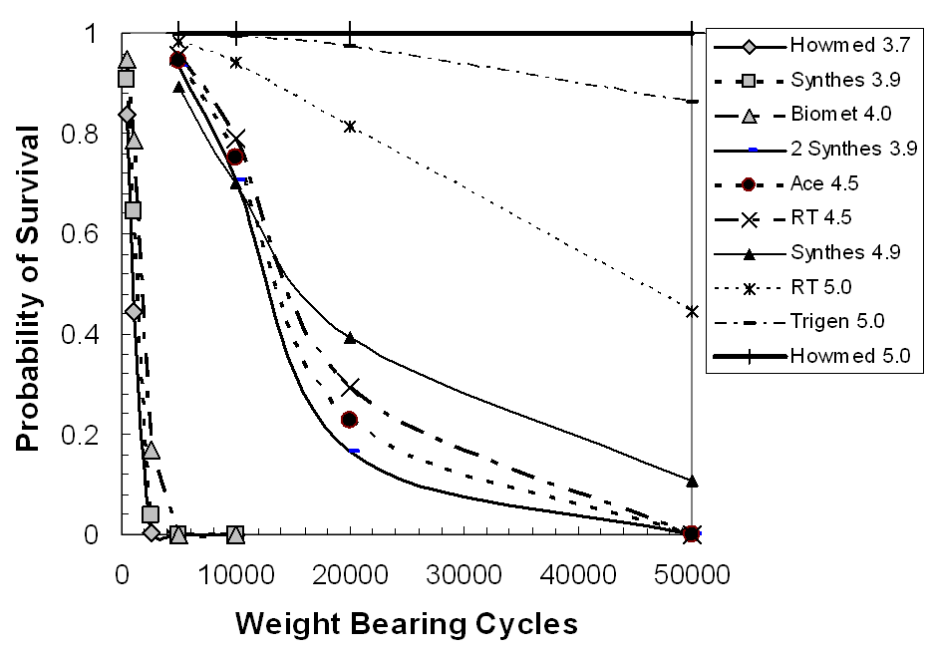
**Probability of survival curves for full weight bearing of the locking screw systems**.

The survival analysis results are shown in Figure 3. All of the small diameter screws have little no chance of lasting a day in a full weight bearing situation. Specifically, the 4.0 mm diameter screw has approximately a 17 percent chance of lasting 2500 cycles (roughly one day), whereas, the 3.7 mm diameter screw survival probability for 2500 cycles is less than a quarter of a percent. For the medium size grouping, the 4.5 mm diameter screws have approximately a 25 percent chance of lasting 20000 cycles (one week), while the two small diameter screws were estimated to have a 17 percent chance of survival. Although this is less, it was not statistically different from the other screws in this group.

**Figure 4:**
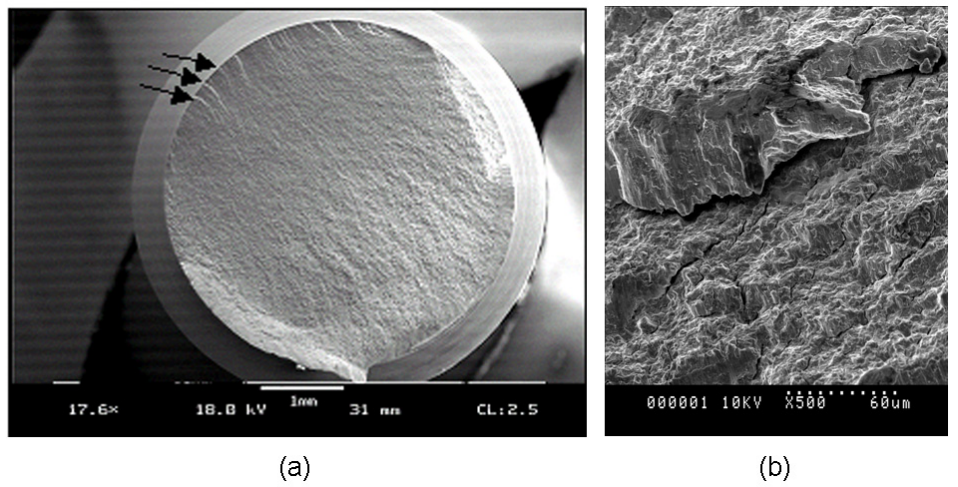
**SEM micrograph showing a typical failure of titanium alloy locking screws**. (a) The arrows indicate crack initiation locations. (b) A view of a rivulet within a crack initiation sight, showing numerous cracks throughout.

**Figure 5:**
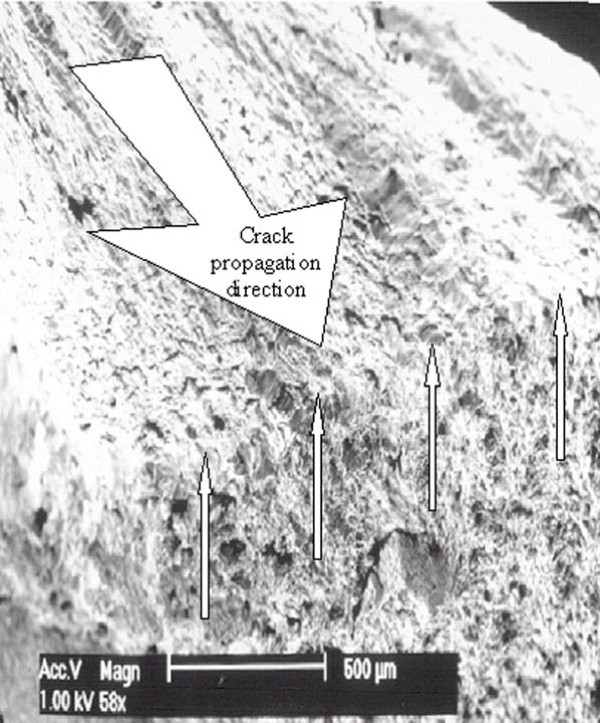
**SEM micrograph showing a typical fatigue failure of a stainless steel locking screw**. The arrows indicate the crack tip location just prior to final fracture occurred.

SEM analyses revealed the failure mode of the titanium alloy is qualitatively different than that of the stainless steel. An SEM image of the Biomet 4.0 mm is shown in Figure 4. The arrows in Figure 4a indicate failure initiation sites. Figure 4b shows a close-up view of a rivulet within an initiation site showing numerous microcracks. Figure 5 is a stainless steel screw fracture surface showing a substantial amount of surface roughness compared to the titanium. Near the center of the micrograph is a demarcation line that separates the fatigue crack growth (smoother) and final fracture (rough).

## Discussion

The purposes of our study were to evaluate the relative fatigue resistance of distal locking screws and bolts from representative manufacturers of tibial IM nail systems, and develop a relative risk assessment of screws and materials used. The development of the locked intramedullary nail has greatly extended the indications for stabilizing the majority of diaphyseal fractures [17]. However, with the evolution and use of smaller unreamed tibial nails with smaller locking screws, the rate of hardware failure has increased. A problem has been failure of the interlocking bolts [16,20]. A potential benefit of the unreamed systems is preservation of the endosteal blood supply. Yet, recent literature has shown no differences in healing rates between reamed and unreamed systems, and larger reamed systems are stiffer with a lower hardware failure rate [20,21].

Our study demonstrates that larger diameter screws (>4.5 mm) show greater fatigue resistance than smaller screws, although there were statistical differences in fatigue life related to material type. For example, the Ace 4.5 mm screw which is made of a titanium alloy has a statistically different fatigue life than the 4.5 mm Russell-Taylor screw, which is made of 316 stainless steel (Table 2, Figure 2). One reason for this is associated with the ductile nature of the stainless steel, which is tougher than the titanium (Figure 5).

**Table 2:** Relative fatigue life of the locking screws

*Manufacturer*	*Diameter (mm)*	*Mean Life*	*St. Dev*
Ace	4.5	10115	4426

Biomet	4.0	1502	615
	
	5.0	360881	469213

Synthes	3.9	1413	490
	
	4.9	16121	3426

Howmedica – Alta	3.7	888	608
	
	5.0	1000000	-^1^

Russell-Taylor	4.5	18238	4009
	
	5.0	46736	13702

S&N – Trigen	5.0	436248	487507

^1 ^None of the Howmedica screws failed and so the St Dev is undefined

**Figure 6:**
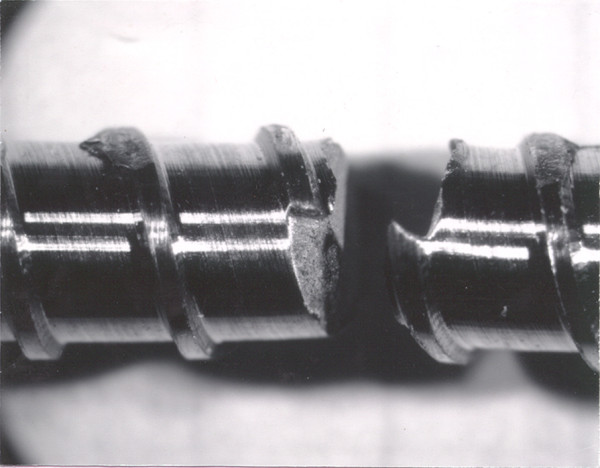
**Optical micrograph showing machining defects caused by thread forming**.

Another potential source of fatigue life variation is thread design and defects from the manufacturing or insertion process. Some of the bolts had obvious surface defects caused by the machining of threads that could act as notches and contribute to the variability of fatigue life by a stress-riser effect (Figure 6). These notches were easily seen under low power microscopy. Notches similar to these machining defects could also be created during deployment of the screw and may also contribute to premature device failure.

Multiple screw configurations profoundly increase the fatigue life of the locking screws by load sharing. In this study, we used two 3.9 mm diameter screws, which offer more cross-sectional area of screw than a 4.5 mm screw, and so it might be expected that 2 screws would last longer than a single 4.5 mm screw. Theoretically, this would be true, but some assumptions need to be made regarding how the loads are shared between the multiple screws, i.e. each screw shares exactly half the load. Practically, this is not true, and so one screw is more heavily loaded than the other, which shortens the life of one of the screws. When one of the screws fails due to fatigue, the entire load is shifted to the other screw; which, in turn, significantly shortens the life of the remaining screw. Therefore, when using multiple screws, it is critical to attempt to distribute the load as uniformly between screws as possible, which can be somewhat challenging, but the effort will lead to a better outcome.

Fatigue is a stochastic process and so it is important to realize that while the average life expectancy and standard deviation has some relevance, the survival analysis is more helpful in that it accounts for the variability. The survival does not assume a statistically normal sample (fatigue and fracture are best represented by a Weibull distribution), and provides a rigorous framework for assessing risk. As an additional confounding factor, the body environment can exert a substantial influence on the results. High stress, corrosion, temperature, and fatigue will act to lower the fatigue life, and so the longer the device is exposed to the body environment, the greater the risk of shortening the fatigue life. Stainless steels are particularly susceptible to the corrosion fatigue process, and so while our tests show that the fatigue life of the stainless steel screws are longer than some similarly sized titanium screws, their fatigue life in the body will be shortened by comparison to the titanium screws.

While we have not included the environmental effects, we have also assumed that all the load is completely carried by the locking bolts, which would neglect any load sharing that occurs due to healing. As healing occurs, the stress on the screws is lowered and the fatigue life increases dramatically. However, if there are complications associated with fracture repair process, it is a matter of time before the locking bolts will fail.

The Howmedica Alta™ system was significantly different from all the other screws we tested. The primary reason for this is the fatigue resistance of the alloy system titanium-molybdenum-zirconium (TMZ) which is stronger than other titanium alloys. Due to the very high strength of this alloy, the ductility of the material is low, which means that the material will tend to behave in a more brittle manner, and may be adversely affected by scratches. The fatigue resistance is highly dependent on diameter, as noted by the results of the 3.7 mm diameter screws (Figure 2, Figure 3). Because the diameter is so small, it is important to use as many screws as possible to ensure the best results.

The length of a fatigue test of a single screw could take many days before failure was reached if we loaded at a physiologic rate of 1 Hz. One million cycles would take about 12.6 days. Therefore, in order to make the study length tractable, we conducted the tests at 20 Hz, and only one of the small screw systems was chosen to do multiple screw fatigue life tests. The higher rate of loading does not adversely affect fatigue life of titanium, and rates approaching 100 Hz are routinely used in high cycle fatigue tests for devices such as stents – in fact, there is evidence that higher frequency loading may slightly lengthen fatigue life [22,23].

## Conclusion

Orthopaedic traumatologists should understand the performance and limitations of the locking bolts used for cases where IM nailing is indicated. This study should aid the selection of the best system for the treatment of the injury. Having a mechanistic understanding of the implant system when coupled with the clinical judgment of the surgeon, can lead to the best functional outcome. Generally speaking, the system that uses the multiple screws and/or the largest distal locking screws would seem to offer the best fatigue resistance for an unstable fracture pattern with a noncompliant patient. While smaller diameter screws are sometimes necessary to use, it is extremely important in those cases to use multiple screws in order to reduce the risk of hardware failure due to fatigue.

## Competing interests

The authors declare that they have no competing interests.

## Authors' contributions

LVG designed and manufactured the fixtures used for the study, designed and participated in the testing, performed materials characterization and the data analysis, assisted in the writing and editing of the manuscript. RMH developed the idea for the study, wrote the protocol, assisted in the data analysis, writing the manuscript, and editing. JJZ performed much of the testing and assisted in the writing and editing of the manuscript.
